# Retroperitoneal Tumors in the Pelvis: A Diagnostic Challenge in Gynecology

**DOI:** 10.3389/fsurg.2014.00049

**Published:** 2014-12-05

**Authors:** Wei-Wei Wee-Stekly, Michael David Mueller

**Affiliations:** ^1^Minimally Invasive Surgery Unit, Division of Obstetrics and Gynecology, KK Women’s and Children’s Hospital, Singapore, Singapore; ^2^University Clinic for Gynecology, Inselspital, Bern, Switzerland

**Keywords:** pelvic retroperitoneal tumors, diagnosis, management, retroperitoneal neoplasms, pelvic mass differential diagnosis

## Abstract

Retroperitoneal tumors can pose a diagnostic and therapeutic challenge to gynecologists because of their rarity, late presentation, and complex anatomical location in the retroperitoneum. This article reviews the diagnosis and management of retroperitoneal tumors in the pelvis, and highlights the potential pitfalls that may be faced by gynecologists.

## Introduction

Retroperitoneal tumors in the pelvis represent a problematic entity of surgical pathology, because of its rarity, difficulty of diagnosis, high recurrence rate after surgical excision, and unpredictable response to adjuvant therapy. Due to the inaccessible anatomical position and late clinical presentation, the diagnosis of retroperitoneal tumors is often made with delay, in the advanced stages of local or systemic spread. As a result, many of these cases do not benefit from complete surgical removal, and this renders a real diagnostic and therapeutic predicament in gynecology.

In this paper, we review the diagnosis and management of retroperitoneal tumors in the pelvis, and highlight the potential diagnostic and therapeutic challenges that may be faced by gynecologists. A literature search was conducted using PubMed. The keywords used for the search included “pelvic retroperitoneal tumors,” “retroperitoneal neoplasms,” “diagnosis,” “investigations,” and “management.” Relevant papers of all article types published in English in the last 10 years were reviewed and selected for inclusion based on relevance to our article.

## Anatomy of the Retroperitoneum

The retroperitoneum represents a complex potential space containing multiple vital structures limited anteriorly by the peritoneum, posteriorly by the posterior abdominal wall, superiorly by the 12th rib and vertebra, inferiorly by the base of the sacrum and iliac crest, and laterally by the borders of the quadratus lumbora muscles. This space contains the connective tissue, kidneys, ureters, adrenal glands, aorta and its branches, inferior vena cava and its tributaries, and lymph nodes (1).

## Types of Retroperitoneal Tumors

Knowing the differential diagnoses of a retroperitoneal tumor will allow the gynecologist to be aware of the necessary pre-operative investigations and referrals so as to optimize the management in the best interest of the patient.

**Figure 1:**
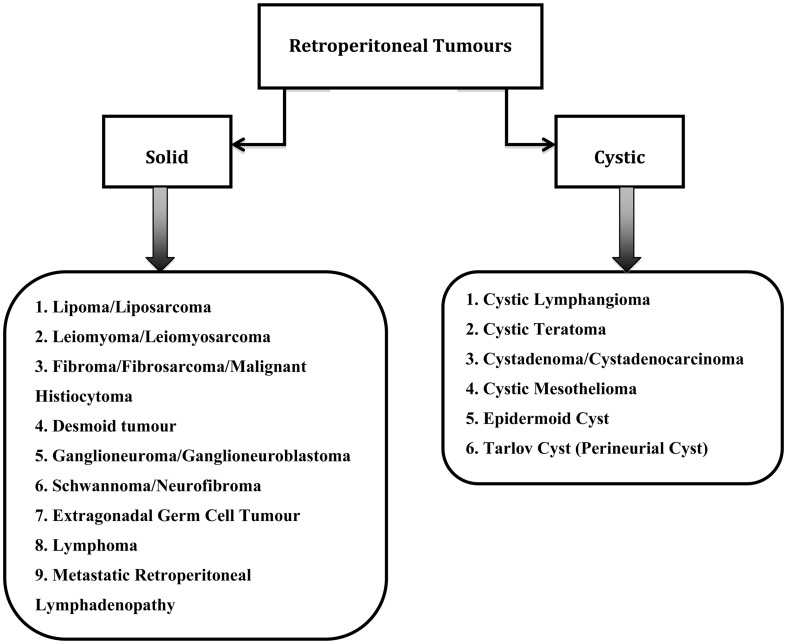
**Types of retroperitoneal tumours**.

Retroperitoneal tumors are rare growths that originate from within the retroperitoneal spaces rather than the major retroperitoneal organs (2). The classification of retroperitoneal tumors can be based on type of tissue origin (3). However in this paper, the retroperitoneal tumors have been divided into solid or cystic tumors as seen in Figure 1. This classification is chosen because gynecologists usually rely on imaging for the diagnosis of retroperitoneal tumors and one of the main radiological descriptions is whether the tumor is solid or cystic. Most retroperitoneal tumors are mesodermal in origin and can arise from any tissue type present in the retroperitoneum. They can be benign or malignant (4).

Benign tumors are often incidental findings during investigations for unrelated symptoms. The most common benign retroperitoneal tumors are schwannomas, neurofibromas, ganglioneuromas, paragangliomas, fibromatosis, and lipomas (5).

Sarcomas make up a third of all the retroperitoneal tumors with predominantly liposarcomas (70%) and leiomyosarcomas (15%) (6). Other malignant retroperitoneal tumors include lymphomas, malignant fibrous histiocytomas (MFH), desmoid tumors, extragonadal germ cell tumors, and metastatic retroperitoneal lymphadenopathy (3).

## Clinical Presentation

Retroperitoneal tumors are commonly misdiagnosed as ovarian pathologies. It is extremely difficult to differentiate a retroperitoneal cyst from an ovarian cyst as the ultrasound findings and clinical symptoms are very similar.

Symptoms secondary to retroperitoneal tumors are vague and appear late in the course of the disease caused by compression of the structures in the retroperitoneum, sometimes causing obstruction. Most patients present with abdominal pain and distension, and will have a palpable mass on clinical examination (7). Many benign retroperitoneal tumors are diagnosed as an incidental finding during imaging for unrelated symptoms (8). Patients can also complain of urinary or gastrointestinal symptoms if there are pressure effects on these organs from the large tumor.

Gatita and colleagues (9) performed a retrospective analysis of all the patients treated for primitive retroperitoneal tumors in their institution between 1999 and 2009. It was interesting to note that all retroperitoneal tumors diagnosed in their study were detected in the symptomatic stage. In this study, 83.9% of the 56 patients presented with abdominal pain, 71.4% presented with a palpable tumor, 51.7% presented with urinary symptoms, 42.9% presented with gastrointestinal symptoms, and 5.4% presented with abdominal distension.

## Diagnosis

The diagnosis of retroperitoneal tumors is made by radiological methods and confirmed by histology. In gynecology, ultrasonography (US) is usually performed as the first line investigation when the patient presents to the consultation clinic with abdominal pain or distension. US is excellent at detecting cystic lesions and is inexpensive to perform with no radiation involved.

The imaging of choice in diagnosing retroperitoneal tumors is contrast-enhanced computed tomography (CT). CT plays a vital role in the localization, characterization, evaluation of the extent of local invasion, assessment of metastases, and determination of treatment response of these tumors (3). Although CT imaging features of most retroperitoneal tumors are non-specific, imaging analysis of the tumor components, growth pattern, vascularity, and tumor demographics can lead to a more precise differential diagnosis (3).

A CT myelography should be performed to rule out Tarlov cyst (perineurial cyst) pre-operatively for every cystic retroperitoneal pelvic tumor suspected on initial imaging, as this will alter the management. It is generally agreed that asymptomatic Tarlov cyst should be left alone; however, symptomatic ones warrant a referral to the neurosurgeons for further management (10, 11).

Albeit more costly than CT, magnetic resonance imaging (MRI) is playing an increasing role in the evaluation of retroperitoneal soft-tissue masses due to its excellent tissue characterization and spatial resolution. However, similar to CT, the MRI features of most retroperitoneal tumors are non-specific, and hence the prediction of a specific histological diagnosis remains a challenge for radiologists. In general, the dynamic enhancement patterns in MRI reflect the vascularity of masses, differentiating benign from malignant retroperitoneal tumors (12). The tissue-specific multiplanar capability of high-resolution MRI allows better tumor localization and internal characterization, thereby serving as a road map for surgical planning (13).

## Management

Complete surgical excision is the treatment of choice for most retroperitoneal tumors. Malignant retroperitoneal tumors like the liposarcomas, leiomyosarcomas, or MFH require wide clear resection margins to allow local control of the disease. The factors affecting complete margin-negative surgical resection include tumor biology, invasion of adjacent structures, surgeon experience, and surgical management in high-volume centers (14–17). Retroperitoneal sarcomas have poor prognosis with a 5-year local recurrence-free survival after complete resection ranging between 55 and 78%, and 5-year overall survival between 39 and 68% (14, 18). Local recurrence is common for retroperitoneal sarcomas and is responsible for as high as 75% of sarcoma-related deaths (18). Surgery should be offered to symptomatic patients with local recurrence as it provides good palliation and improves the survival rate for selected patients (19). Palliative debulking surgery for recurrent retroperitoneal sarcomas of low and intermediate grade can be offered to patients for symptom control and may improve their quality of life (20).

Benign retroperitoneal tumors such as the schwannomas, neurofibromas, or Tarlov cysts will have to grow to a significant size before becoming palpable or symptomatic. Management options for these benign tumors include radiological surveillance in asymptomatic patients or surgical resection in symptomatic patients (8, 10, 11).

Chemotherapy is the mainstay treatment for the Ewing family of tumors (21). Chemotherapeutic agents such as doxorubicin and ifosfamide have also been used in the palliation of symptomatic advanced retroperitoneal sarcoma (21). There is increasing evidence to support the use of specific chemotherapeutic agents to target certain histological subtypes. For instance, the use of gemcitabine and docetaxel for leiomyosarcoma, taxanes for angiosarcoma, and trabectedin for myxoid/round cell liposarcoma and leiomyosarcoma (21). If distant metastasis is present, post-operative adjuvant chemotherapy will also be required to contain the disease.

Till date, the appropriate role, dose, and timing of radiotherapy in the management of retroperitoneal sarcoma are still not established due to the lack of randomized controlled trials. Retroperitoneal sarcomas are often not amenable to conventional radiotherapy alone (14, 15, 18). Combined modality treatment (combination of surgery with radiotherapy) has been proven to improve the local recurrence rate in retroperitoneal sarcomas (22). Several observational and retrospective studies have been published to evaluate the outcomes of pre-, intra-, and post-operative radiotherapy in the management of retroperitoneal sarcomas (23–26). Pre-operative radiotherapy offers these advantages: a clear demarcated area of tumor for radiotherapy planning, the displacement of the adjacent radiosensitive structures by the tumor, and the equivalent therapeutic dose of radiotherapy may be lower in the pre-operative setting. Post-operative radiotherapy allows the selection of patients at highest risk of recurrence based on the surgical margins and histological grade. On the down side, in the post-operative setting, the adjacent structures will occupy and become adherent to the tumor bed, increasing the risk of radiation associated toxicities. Therefore, the use of intra-operative radiotherapy or intensity-modulated radiation therapy has been investigated in an attempt to reduce the radiation toxicities (24, 26).

An important development in surgery has been the concept of concentrating rare surgical conditions and complex operations in high-volume specialist centers (27). Gutierrez and colleagues (17) analyzed 4205 patients treated for retroperitoneal tumors in their study comparing the outcome between low and high-volume centers. The authors concluded that patients with large, high-grade, especially retroperitoneal tumors should be treated exclusively in high-volume centers to ensure improved short-term surgical outcomes, lower local recurrence rate, and superior overall survival rate (17). Therefore, the management of retroperitoneal tumors should be limited to a few dedicated, experienced multidisciplinary centers with the necessary expertise, not just to optimize patient care, but also to benefit training and research. Being a rare and complex tumor, retroperitoneal sarcomas should be managed by an experienced multidisciplinary team in a specialized sarcoma center (28).

Research into the tumor biology of retroperitoneal sarcomas shows promise of the development of novel biological therapies to target the various molecular pathways (29). This avenue of treatment will broaden the management options in future, especially for patients not fit for surgery.

## Diagnostic and Therapeutic Challenge

In addition to the inaccessibility of the retroperitoneal region, retroperitoneal tumors often show no or non-specific symptoms until they have grown to a substantial size. As a result, they are usually very large at presentation. In other words, the clinical manifestations of retroperitoneal tumors are variable and only reveal themselves after a long latency period when the tumors are large enough to cause compression, displacement, or invasion of adjacent structures. This late diagnosis is not optimal as the prognosis of a malignant retroperitoneal tumor is influenced by the time of diagnosis.

The diagnosis is also not straightforward as there are many visceral and vascular structures located in the pelvis and retroperitoneal space. Often on US, a retroperitoneal tumor is mistaken for a gynecological pelvic tumor, only to be proven wrong during surgery. Therefore, CT and MR imaging play a vital role in the diagnosis of a retroperitoneal tumor. However, it is still a diagnostic challenge as many of the imaging features are non-specific for a particular tumor. Hence, a definitive diagnosis can only be established at histopathological examination.

In addition to the above mentioned diagnostic dilemmas, retroperitoneal sarcomas pose several therapeutic challenges. These tumors have variable radiosensitivity, and are often adjacent to radiosensitive structures with low radiation tolerance in the retroperitoneum.

Being in an anatomically complex and surgically inaccessible site with surrounding vital structures, it may not always be possible to obtain a complete resection of the retroperitoneal tumor with sufficient clear margins, especially when most tumors are large at presentation, hence compounding the technical difficulty of the surgery. Inexperienced surgeons may find it challenging to operate on a tumor in the complex retroperitoneum. It is essential for surgeons to have sound knowledge of the anatomy of the retroperitoneal space in order to avoid injury to the adjacent visceral, vascular, and nervous structures, resulting in complications like hemorrhage and neurological deficits.

## Conclusion

There is a wide spectrum of rare tumors in the retroperitoneum, both benign and malignant. The presentation of these tumors is usually late; patients commonly present with symptoms of abdominal pain or a palpable tumor. CT and MR imaging play an important role in the diagnosis of retroperitoneal tumors. However, as the imaging features are non-specific, the diagnosis can only be confirmed by histology. Complete surgical resection is the mainstay treatment when a patient is symptomatic. A benign retroperitoneal tumor can be treated conservatively with frequent radiological surveillance if the patient is asymptomatic. In the case of retroperitoneal sarcomas, this is best managed in a dedicated, experienced multidisciplinary sarcoma center. It is beneficial to gynecologists to be aware of the management of retroperitoneal tumors and its pitfalls.

## Conflict of Interest Statement

The authors declare that the research was conducted in the absence of any commercial or financial relationships that could be construed as a potential conflict of interest.
